# Vehicle recognition pipeline via DeepSort on aerial image datasets

**DOI:** 10.3389/fnbot.2024.1430155

**Published:** 2024-08-16

**Authors:** Muhammad Hanzla, Muhammad Ovais Yusuf, Naif Al Mudawi, Touseef Sadiq, Nouf Abdullah Almujally, Hameedur Rahman, Abdulwahab Alazeb, Asaad Algarni

**Affiliations:** ^1^Faculty of Computing and AI, Air University, Islamabad, Pakistan; ^2^Department of Computer Science, College of Computer Science and Information System, Najran University, Najran, Saudi Arabia; ^3^Department of Information and Communication Technology, Centre for Artificial Intelligence Research, University of Agder, Grimstad, Norway; ^4^Department of Information Systems, College of Computer and Information Sciences, Princess Nourah Bint Abdulrahman University, Riyadh, Saudi Arabia; ^5^Department of Computer Sciences, Faculty of Computing and Information Technology, Northern Border University, Rafha, Saudi Arabia

**Keywords:** deep learning, remote sensing, object recognition, unmanned aerial vehicles, DeepSort, dynamic environments, path planning

## Abstract

**Introduction:**

Unmanned aerial vehicles (UAVs) are widely used in various computer vision applications, especially in intelligent traffic monitoring, as they are agile and simplify operations while boosting efficiency. However, automating these procedures is still a significant challenge due to the difficulty of extracting foreground (vehicle) information from complex traffic scenes.

**Methods:**

This paper presents a unique method for autonomous vehicle surveillance that uses FCM to segment aerial images. YOLOv8, which is known for its ability to detect tiny objects, is then used to detect vehicles. Additionally, a system that utilizes ORB features is employed to support vehicle recognition, assignment, and recovery across picture frames. Vehicle tracking is accomplished using DeepSORT, which elegantly combines Kalman filtering with deep learning to achieve precise results.

**Results:**

Our proposed model demonstrates remarkable performance in vehicle identification and tracking with precision of 0.86 and 0.84 on the VEDAI and SRTID datasets, respectively, for vehicle detection.

**Discussion:**

For vehicle tracking, the model achieves accuracies of 0.89 and 0.85 on the VEDAI and SRTID datasets, respectively.

## 1 Introduction

Rapid economic and demographic expansion generate a dramatic surge in vehicle numbers on highways. Hence, complete road traffic monitoring is necessary for acquiring and evaluating data, essential for comprehending highway operations within an intelligent, autonomous transportation framework (Dikbayir and İbrahim Bülbül, 2020; Xu et al., 2022; Yin et al., 2022). Consequently, there’s a compelling need to automate traffic monitoring systems. While various image-based solutions have been developed, obstacles exist in expanding their capabilities, especially in dynamic contexts where backdrop and objects are in flux (Weng et al., 2006; Di et al., 2023; Dai et al., 2024). Traditional approaches like background removal and frame differencing struggle when used to photographs acquired from mobile platforms owing to background motion, blurring the boundaries between background and foreground objects. Hence, improvements in computer vision and image processing, covering disciplines such as intelligent transportation, medical imaging, object identification, semantic segmentation, and human-computer interaction, present promising paths (Angel et al., 2003; Cao et al., 2021; Ding et al., 2024).

Semantic segmentation, defining and identifying pixels by class, provides a sophisticated method (Schreuder et al., 2003; Sun et al., 2020; Ren et al., 2024). Unlike current systems confined to binary segmentation (e.g., vehicle vs. backdrop), our suggested technique utilizes multi-class segmentation, expanding scene knowledge (Ding et al., 2021; Gu et al., 2024). Moreover, utilizing aerial data offers enormous promise in boosting traffic management. However, obstacles such as varying item sizes, wide non-road regions, and different road layouts need efficient solutions to exploit mobile platform-derived data effectively (Najiya and Archana, 2018; Sun et al., 2018; Omar et al., 2021).

In this study, a unique approach for the identification and tracking of vehicles is based on aerial images. In our approach, aerial films are first transformed into frames for images (Sun et al., 2023). Defogging and gamma correction methods are then used for noise reduction and bright-ness improvement, respectively, while pre-processing is being done on those frames (Qu et al., 2022; Chen et al., 2023a; Zhao X. et al., 2024). After that, Fuzzy C Mean and DBSCAN algorithm is used for segmentation to decrease the background complexity. YOLOv8 is applied for recognition of automobiles in each extracted frame as it can detect tiny objects successfully. To track several cars inside the image’s frames, all identified vehicles have been allocated an ID based on ORB attributes. Also, to estimate the traffic density on the roadways, a vehicle count has been kept throughout the picture frames. The tracking has been done using the DeepSort with Kalman filter. Moreover, the provided traffic monitoring systems were verified by the tests done on VEDAI, and SRTID datasets. The studies have exhibited amazing detection and tracking precision when compared to other state-of-the-art (SOTA) approaches.

Some of the prominent contributions of this work include:

- Our model reduces model complexity by combining pre-processing methodologies with segmentation techniques for the preparation of frames prior to detection phase.
- Evaluation of unsupervised segmentation strategies, specifically Fuzzy C-Mean (FCM) algorithm and Density-Based Spatial Clustering of Applications with Noise (DBSCAN), was undertaken, boosting segmentation effectiveness.
- Significantly enhanced accuracy, recall, and F1 Score in vehicle recognition and tracking compared to earlier techniques have been obtained.
  Implementation of vehicle tracking leveraging the DeepSort algorithm, reinforced by an ID assignment and recovery module based on ORB, has been successfully accomplished, exhibiting remarkable performance proven across two publicly available datasets.

The article is structured into the following sections: Section 2 dives into the literature on traffic analysis. Section 3 goes over the proposed technique in great depth. Section 4 describes the experimental setting, offering empirical insights into the system’s performance. Section 5 reviews the system’s performance and considers its advantages and disadvantages. Section 6. Discuss the work’s limitations. Section 7 is the conclusion, which summarizes the main results and proposes future research and development goals.

## 2 Literature review

Over the last several years, academics have aggressively excavated into constructing traffic monitoring systems. They have examined the behaviors of their systems utilizing multiple picture sources, including static camera feeds, satellite images, and aerial data (Li J. et al., 2023; Wu et al., 2023). Typically, the full photos undergo first preprocessing to exclude non-essential components beyond cars, followed by feature extraction (Hou et al., 2023a). Different strategies depend on techniques such as image differencing, foreground extraction, or background removal, especially when the Region of Interest (ROI) is well-defined and suitably sized within the images (Shi et al., 2023; Zhu et al., 2024). Aerial imaging can cause the size of vehicles to vary based on the height of image acquisition. Because of this, semantic segmentation techniques have become popular for detection and tracking applications. It is also common to use additional stages such as clustering and identifier assignment to improve results. Deep learning algorithms have become popular in recent years for object recognition, showing better performance in handling complex situations (Wang et al., 2024; Yang et al., 2024). To provide an overview of current models and approaches, the linked research is classified into machine-learning and deep learning-based traffic system analyses.

### 2.1 Machine learning-based traffic scene analysis

Machine learning has been extensively used in computer vision-related jobs for a long time, particularly in traffic control and monitoring. To find the cars in the images (Rafique et al., 2023), introduced a vehicle recognition model based on haar-like characteristics with an AdaBoost classifier. In Drouyer and de Franchis (2019), a method for monitoring traffic on highways using medium resolution satellite images is shown. The backdrop image difference approach was used to identify the items in motion after a median filter was applied to the images after road masking for the elimination of irrelevant regions. Next, the gray level of the resultant image was computed. The last phase used a thresholding strategy to identify large, bright spots as autos. According to the authors in Hinz et al. (2006), motion detection algorithms are in-effective because aerial images include motion in both the foreground and background. Therefore, an approach based on morphological operations, the Otsu partitioning method, and bottom-hat and top-hat transformations was applied for detection. After extracting the Shi Tomasi features, clusters were formed based on displacement and angle trajectories. The automobiles vanished behind the backdrop clusters. Each vehicle’s robust feature vector was used for tracking. To achieve excellent precision, they used several feature maps. Vehicle detection has been accomplished utilizing two distinct methods in separate research (Chen and Meng, 2016). While the other approach employed HSV color characteristics in conjunction with the Gray Level Co-occurrence Matrix (GLCM) to identify cars, the first methodology used features from the Accelerated Segment Test (FAST) and HOG features. Vehicle tracking is achieved via the use of Forward and Backward Tracking (FBT).

The background subtraction approach is used by Aqel et al. (2017) to identify moving automobiles. Morphological adjustments are carried out to reduce the incidence of false positives. Ultimately, the invariant Charlier moments are used to achieve categorization. The method’s applicability to a variety of traffic circumstances is limited by the usage of standard image processing methods. Additionally, the automobiles that are not moving will be removed using the background subtraction approach, which will lower the true positives. Another traffic monitoring strategy has been provided by Mu et al. (2016). The model selected the area with a high Absolute Difference (SAD) value as a moving vehicle after computing the image difference. Vehicles have been found and matched across many picture frames using SIFT. The authors of Teutsch et al. (2017) used a novel technique for stacking images. The image registration process was limited to tiny autos, and the warping approach was used to blur any stationary backdrops close to moving vehicles. The main goal of this algorithm is to remove distracting backdrop features from images so that only the vehicle is visible when the surrounding region is smoothed out. These systems have a high temporal complexity, and these approaches were distinguished by their complicated properties. These methods incur high computational costs. Furthermore, the model’s generalizability is weakened as scene complexity rises.

### 2.2 Deep learning-based traffic scene analysis

Traffic monitoring has always included manual techniques and in-car technology. Nonetheless, deep learning is more effective than traditional methods when it comes to image processing. An automobile recognition method based on the You Look Only Once (YOLOv4) deep learning algorithm has been presented by Lin and Jhang (2022). Another study Bewley et al. (2016) employed the Faster R-CNN as the target detector and developed a tracking method (SORT) for real-time systems based on the Hungarian matching algorithm and the Kalman filter to track several targets at once. The SORT tracker does not take the monitored object’s appearance characteristics into account. A technique for detecting automobiles using an enhanced YOLOv3 algorithm is proposed by Zhang and Zhu (2019). To increase the detection method’s accuracy, a new structure is added to the pre-trained YOLO network during training. YOLOv3, on the other hand, is among the most ancient. Using the most recent designs may enhance the detection result. Miniature CNN architecture, as described by Ozturk and Cavus (2021), is a vehicle identification model that relies on Convolutional Neural Networks (CNNs) in conjunction with morphological adjustments. The computational cost of this post-processing is high. Moreover, different aerial image databases show different levels of accuracy. A method for real-time object tracking and detection was reported by the authors in Alotaibi et al. (2022). An enhanced RefineDet-based detection module is included in the model. Additionally, the twin support vector machine model and the harmony search technique are employed for classification. Pre-processing of the data is absent from the model, which might lower the model’s total computing complexity. A vehicle identification model based on deep learning is shown in Amna et al. (2020). Convolutional Neural Networks (CNN) are used by the model to recognize vehicles, while radar data is used to determine the target’s location. A two-stage deep learning model is developed in different research (He and Li, 2019). In addition to detecting cars, the model also recognizes them again in subsequent frames, which is a crucial component of tracking. As opposed to traditional appearance and motion-based characteristics, the re-identification is mostly reliant on vehicle tracking context information.

There is always room for development in the field of automated traffic monitoring systems, despite the substantial research that has been done in this area. To get effective results, efficient and specialized designs are needed for the recognition of automobiles in aerial images, particularly in situations with heavy traffic. Machine learning techniques are insufficient to distinguish between objects whose pixels exhibit motion. As a result, we use deep learning strategies to raise the precision of vehicle tracking and detection.

## 3 Materials and methods

### 3.1 System methodology

**Figure 1:**
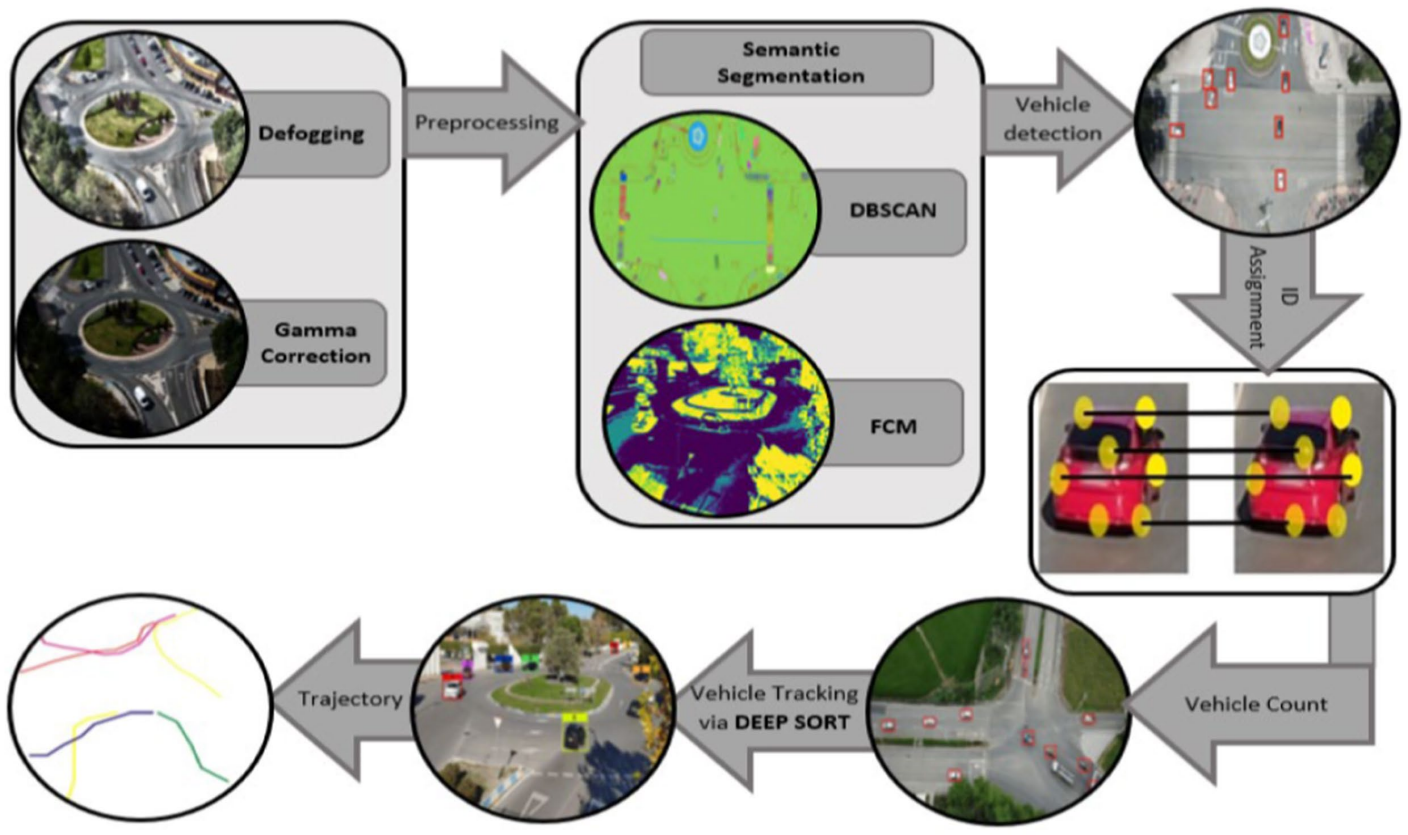
Flowchart demonstrating the proposed traffic surveillance system proposed system architecture.

This section details the planned traffic monitoring system. System architecture overview is provided in Figure 1. This work offers a vehicle recognition and tracking system based on semantic segmentation. Firstly, the videos are turned into frames and pre-processing processes, i.e., defogging for noise reduction are done to the images. Then Gamma correction is employed for adjustment of image intensity for enhanced detection. FCM and DBSCAN segmentation was done on the filtered images for separation of foreground and back-ground items. YOLOv8 is applied for vehicle detection. ORB attributes are used for the assignment of unique ID. Vehicles were traced over several frames of images using a Deepsort. For finding each tracked vehicle, ORB key point description combined with trajectories approximation are used to recover IDs. Further information on each module is given in the ensuing subsections.

### 3.2 Images pre-processing

**Figure 2:**
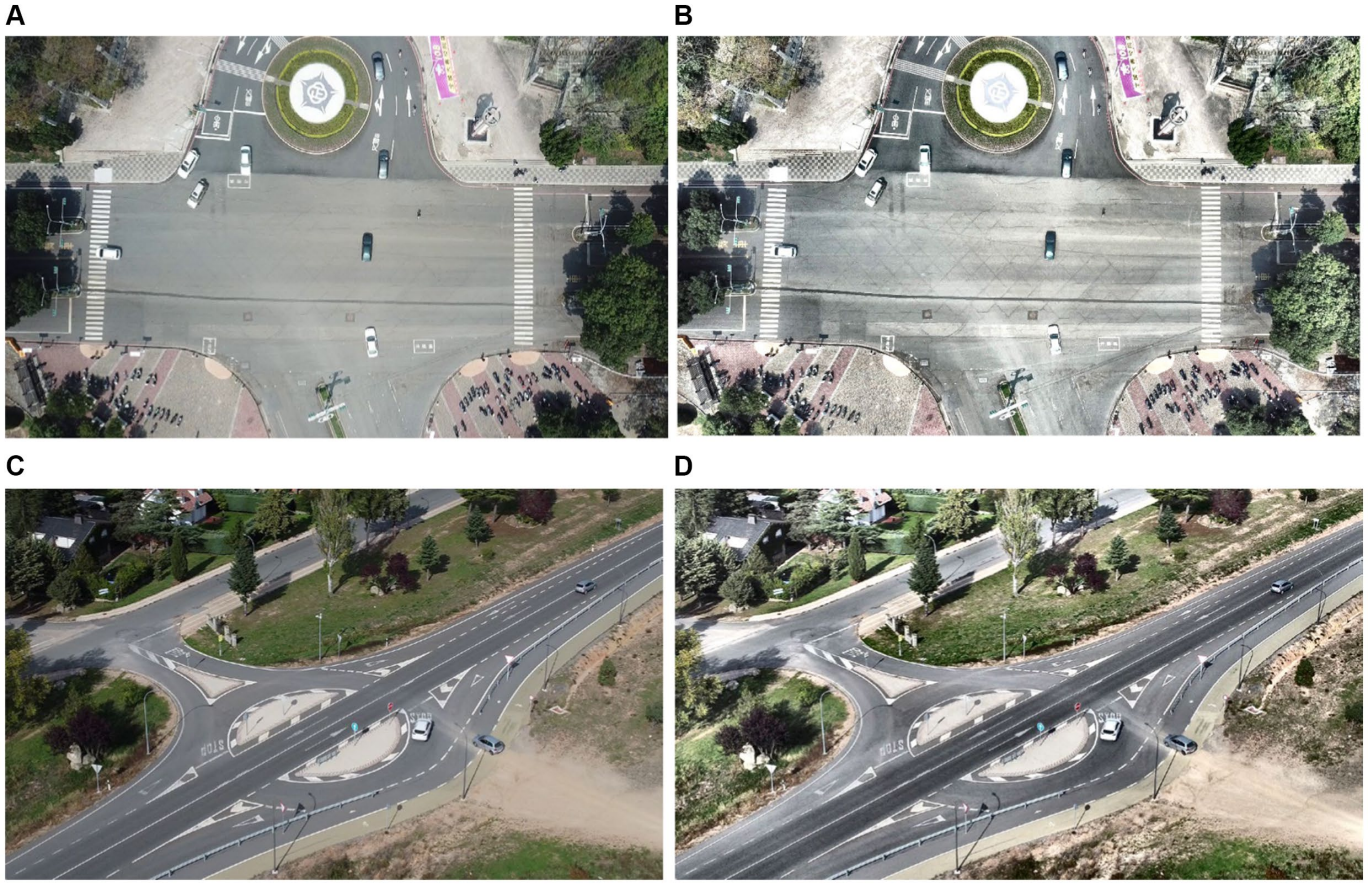
Defogging results over the **(A)** original image of VEDAI dataset **(B)** defogged image **(C)** original image of SRTID dataset **(D)** defogged image.

**Figure 3:**
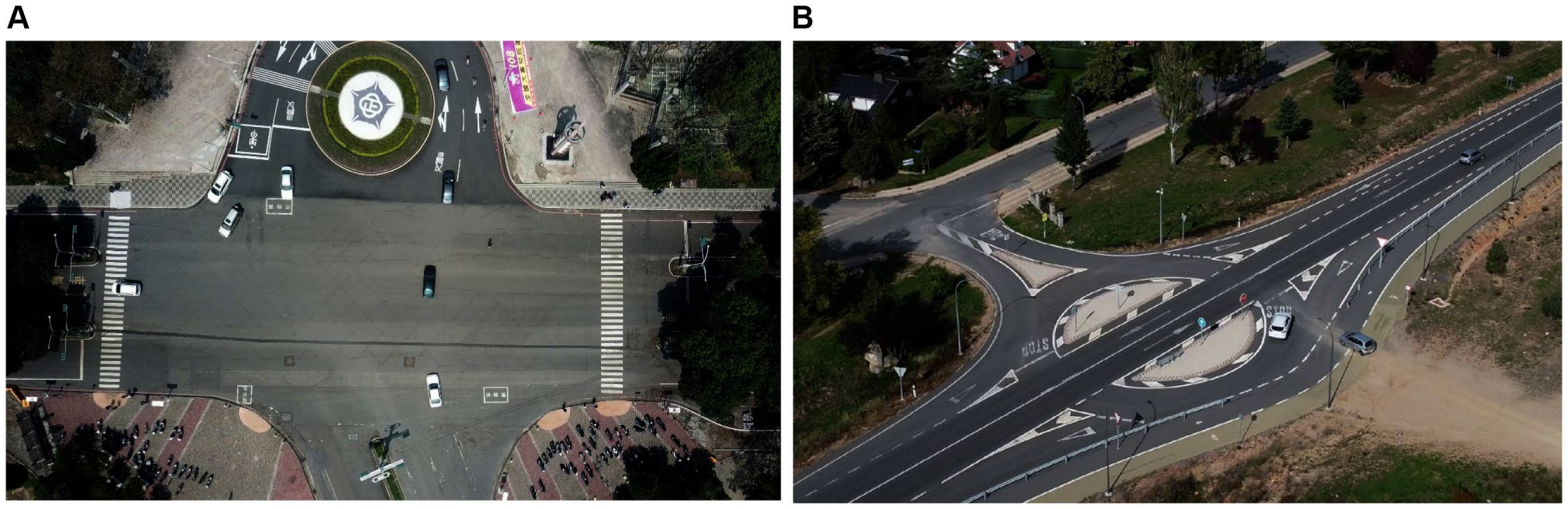
Pre-processed image using gamma correction over the **(A)** VEDAI dataset **(B)** SRTID dataset.

To eliminate superfluous pixel information from the resulting image, noise reduction is necessary since the extra pixel’s complicate recognition (Rong et al., 2022; Xiao et al., 2023). For best performance, any filter using defogging methods is applied to noise (Gao et al., 2020; Tang et al., 2024). The defogging technique measures the amount of noise in each pixel of the picture and then removes it in the following ways.


$$G\left(x\right)=X\left(x\right)Y\left(x\right)+Z\left(1-p\left(x\right)\right)$$

where pixel location is denoted by *x*, fog density by *Z*, and transmission map by *Y*(*x*). Figure 2 represents defogged images:

The denoised image’s intensity is then adjusted using gamma correction (Huang et al., 2018; Zhao L. et al., 2024) since a high brightness allows for the most effective detection of the area of interest. The gamma correction power-law is provided as follows:


$$Vo=TV_{I}^{\gamma }$$

where *V_I_* is the non-negative value with power *γ* of the input, which may vary from 0 to 1, and *T* is a constant, usually equals to 1. *V_o_* stands for the final image. The plotted denoised, intensity adjusted. Figure 3 shows the gamma-corrected images.

### 3.3 Semantic segmentation

In many computer vision applications, including autonomous vehicles, medical imaging, virtual reality, and surveillance systems, image segmentation is essential. Images are divided into homogeneous sections using segmentation methods. Every area stands for a class or object. To improve item recognition on complicated backdrops, we compared two segmentation techniques.

#### 3.3.1 FCM segmentation

Segmentation is widely employed in a variety of computer vision applications. This is a fundamental stage. Segmentation methods separate images into homogeneous sections (Huang et al., 2019; Hao et al., 2024). Each area denotes an item or class. We used the Fuzzy C-Mean segmentation technique. FCM is a clustering method in which each picture pixel might belong to two or more groups. Fuzzy logic (Chong et al., 2023; Zheng et al., 2024) refers to pixels that belong to more than one cluster. Because we are working with many complicated road backdrops including several items and circumstances, segmentation approaches based on explicit feature extraction and training are unable to deliver a generic solution. For this purpose, we used FCM, a non-supervised clustering algorithm. During the FCM segmentation process, the objective function is optimized across numerous rounds. Throughout the iterations, the clustering centers and membership degrees were continually updated (Rehman and Hussain, 2018). The FCM method separates a finite collection of N items (S=𝑠1, 𝑠2, 𝑠𝑛) into C clusters. Each component of *𝑣𝑖* (i = 1, 2…, *N*) is a vector of d dimensions. We design a technique to divide s into C clusters using cluster centers 𝑢1, 𝑢2, and so on in the centroid set u (Xiao et al., 2024; Xuemin et al., 2024). The FCM approach uses a representative matrix (g) to represent the membership of each element in each cluster. The matrix 𝑔 may be defined using equation:


$$g\left(i,z\right),1\leq i\leq N;\;1\leq z\leq C$$

where *𝑔 (𝑖, 𝑧)* represents the membership value of the element 𝑠𝑖 having cluster center 𝑣𝑧. While calculating performance index *J_fcm_*, and it is used to calculate the weighted sum of the distance between cluster center and components of the associated fuzzy cluster.


$$\mathrm{Jfcm}=\left(g,u\right)=\overset{M}{\underset{i=1}{\sum }}\overset{N}{\underset{Z=1}{\sum }}g_{iz}^{b}\;{\left|\left|s_{i}-v_{z}\right|\right|}^{2},1<b<\infty$$

where *m* indicates the number of clusters, *N* signifies the number of pixels, *𝑠𝑖* is the *𝑖*𝑡ℎ pixel, *𝑣𝑧* is the *𝑧*tℎ cluster center, and 𝑏 represents the blur exponent. The degree of membership function must meet the conditions specified in the equation below.


$$0\leq b_{iz}\leq 1\;\overset{r}{\underset{z=1}{\sum }}b_{iz}=1,\overset{M}{\underset{i=1}{\sum }}b_{iz}\leq M,i=1,2,..M\;\mathrm{and}\;z=1,2,..,r$$

Each time the membership function matrix is updated using equation:


$$b_{iz}^{b}=\frac{1}{\sum_{j=1}^{c}{\left(\frac{d_{iz}^{2}}{d_{jz}^{2}}\right)}^{\frac{2}{b-1}}}$$

The membership matrix ( $b_{iz}^{b}$ ) is between [0,1], and the distance between cluster centroid (𝑣𝑖) and pixel (𝑠𝑧) is supplied by $\left(d_{iz}^{2}\right)$ . The cluster centroid is determined by equation:


$$v_{j}=\frac{\sum_{z=1}^{N}g_{iz}^{b}s_{z}}{\sum_{z=1}^{N}g_{iz}^{b}}$$

**Figure 4:**
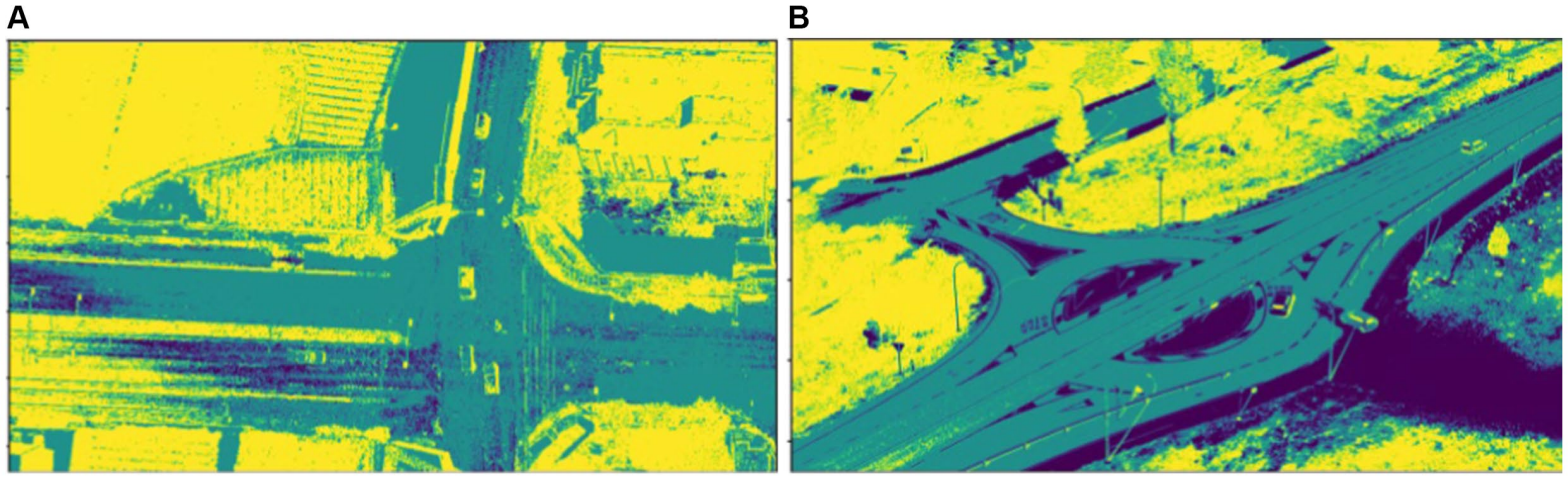
Segmentation using FCM over **(A)** VEDAI dataset and **(B)** SRTID dataset.

A pixel receives a high membership value as it gets closer to the belonging cluster center and vice versa. Figure 4 depicts the results of the FCM segmentation.

#### 3.3.2 Density-based spatial clustering (DBSCAN)

**Figure 5:**
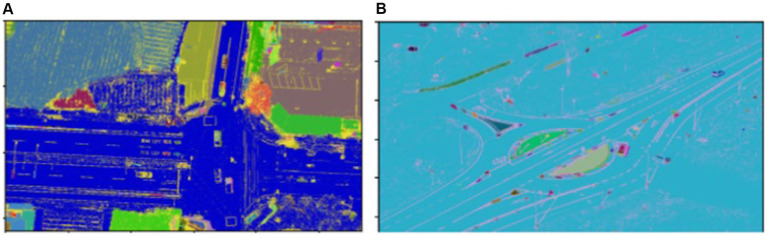
Segmentation using DBSCAN over **(A)** VEDAI dataset and **(B)** SRTID dataset.

DBSCAN, or density-based spatial clustering, is a popular method in machine learning and data analysis (Khan et al., 2014; Deng et al., 2022). In contrast to conventional clustering techniques that need preset cluster numbers, DBSCAN utilizes a data-centric methodology. It uses data density and closeness to its advantage to detect variable-sized and irregularly formed clusters within complicated datasets (Bhattacharjee and Mitra, 2020; Liu et al., 2023). Initially, core points are determined based on having the fewest surrounding data points within a certain distance. These core locations are then expanded into clusters by adding nearby data points that satisfy density requirements (Chen et al., 2022; Zhang et al., 2023). Noise is defined as any data point that does not fit into a designated cluster or core point.


$$N_{\varepsilon }\left(x_{i}\right)=\;\left\{x_{j}\in X|\mathrm{dist}\left(x_{i},x_{j}\right)\leq \varepsilon \right\}$$

where $N_{\varepsilon }\left(x_{i}\right)$ represent the neighborhood of a point $x_{i}$ , $x_{j}\in X$ denotes all points $x_{j}$ belonging to the dataset X , $\mathrm{dist}\left(x_{i},x_{j}\right)$ calculates the distance between points, ε is a threshold distance parameter, defining the maximum distance for points to be considered neighbors (see Figure 5).


$$C=\;\left\{x_{i}\in X||N_{\varepsilon }\left(x_{i}\right)\geq \mathrm{MinPts}\right\}$$

$$x_{i}\in X||N_{\varepsilon }\left(x_{j}\right)\;\mathrm{and}\;x_{j}\in C\text{,}X=\;\left\{x_{1,}x_{2,\ldots ..}x_{n}\right\}\text{.}$$

where $x_{i}$ is the epsilon neighborhood of $x_{j}$ and $x_{j}$ is the core point.

The FCM and DBSCAN segmentation methods were evaluated in terms of computational cost and error rates determined using equations.


ErrorRate=1−accuracy

FCM surpasses DBSCAN owing to its adeptness in managing datasets with varied cluster shapes and sizes. By adding fuzzy membership degrees, FCM addresses the ambiguity inherent in data point assignments, resulting in more adaptive and improved clustering. Furthermore, FCM enables increased control over cluster boundaries via parameterization, allowing for exact alterations to better fit the specific properties of the data. Table 1 exhibits FCM’s better efficacy and accuracy in picture segmentation on VEDAI and SRTID datasets. Considering both computation time and error rates, FCM shines, making its findings the preferable option for following tasks such as vehicle recognition, ID allocation, recovery, counting, and tracking.

**Table 1:** Error rate comparison of DBSCAN and FCM.

Datasets	Error rate	
	DBSCAN	FCM
VEDAI	0.32	0.20
SRTID	0.37	0.23

### 3.4 Vehicle detection

YOLOv8 is utilized for vehicle recognition and radiates as an excellent single-shot detector capable of identifying, segmenting, and classifying with fewer training parameters (Chen et al., 2023b; Wang et al., 2023). According to the CSP principle, the C2f module replaces the C3 module to align with the YOLOv8 backbone, increasing gradient flow information while keeping YOLOv5 compliant. The C2f module combines C3 with ELAN in a unique manner, drawing on YOLOv7’s ELAN methodology, ensuring YOLOv8 compatibility (). The SPPF module at the backbone’s end employs three consecutive 5 × 5 Maxpools before concatenation in each layer to reliably identify objects of varied sizes with lightweight efficiency (Sun et al., 2019; Li S. et al., 2023; Yi et al., 2024).

**Figure 6:**
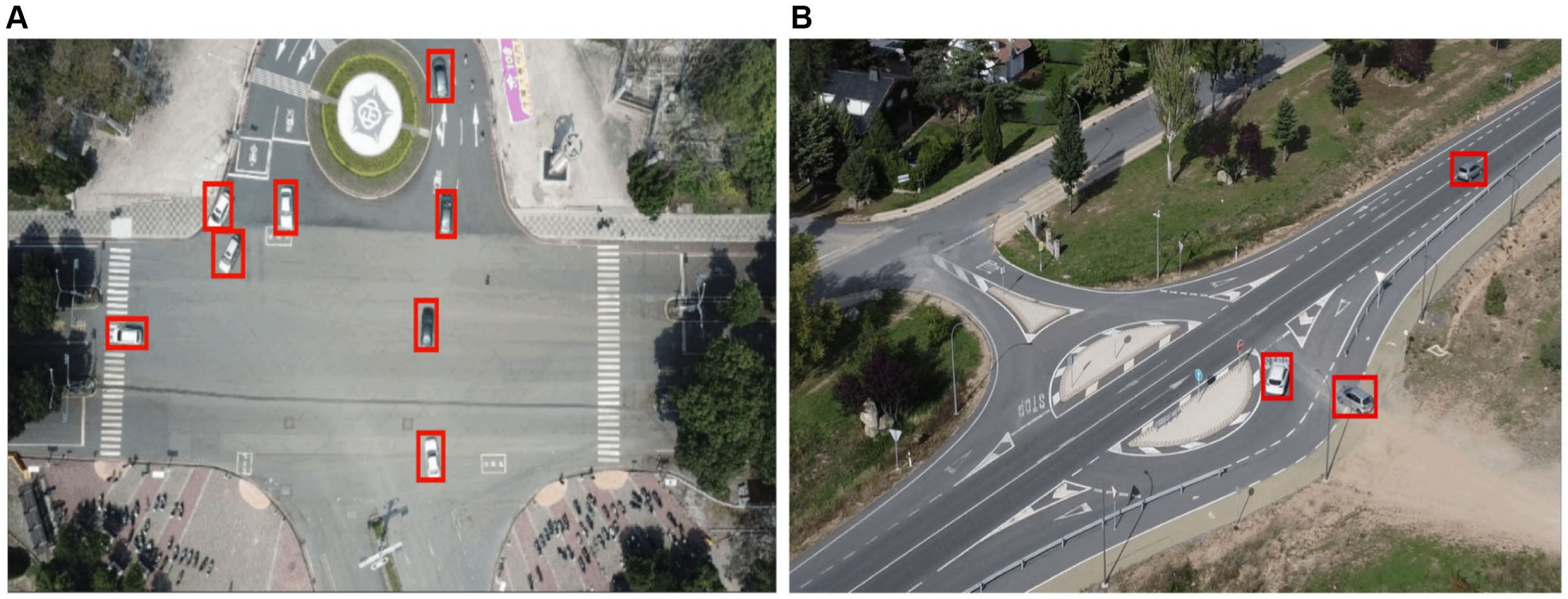
Vehicle Detection over **(A)** VEDAI and **(B)** SRTID datasets marked with red boxes via the YOLOv8 algorithm.

YOLOv8 integrates PAN-FPN in its neck portion, which improves feature fusion and data use at different sizes (Mostofa et al., 2020 Xu et al., 2020). The neck module combines a final decoupled head structure, many C2f modules, and two up samplings (Song et al., 2022; Wu and Dong, 2023). YOLOv8’s neck is like YOLOx’s head idea, which combines confidence and regression boxes to increase accuracy. It operates as an anchor-free model, detecting the object center directly, lowering box predictions, and speeding up the Non-Maximum Suppression (NMS) process, an important post-processing step (Li et al., 2024). Figure 6 shows automobiles spotted using YOLOv8.

### 3.5 ID allocation and recovery based on ORB features

Prior to tracking each identified vehicle in the subsequent image frames, an ID based on ORB traits was assigned to each detected vehicle. A quick and effective feature detector is ORB (Chien et al., 2016; Chen et al., 2022). FAST (Features from Accelerated Segment Test) key point detector is used for key-point detection. It is a more sophisticated version of the BRIEF (Binary Robust Independent Elementary Features) description. It is also rotationally and scale-invariant. Equation is used to get a patch moment (Luo et al., 2024; Yao et al., 2024).


$$n_{st}=\sum x^{s}y^{t}l\left(u,v\right)$$

where *x* and *y* are the image pixels’ relative intensities, represented by the values *s* and *t*. These moments may be utilized to find the center of mass using equation:


$$N=\frac{m_{10}}{m_{00}},\frac{m_{01}}{m_{00}}$$

where the equation defines path orientation:


$$\theta =\mathrm{atan}\;\left(m_{01},m_{10}\right)$$

**Figure 7:**
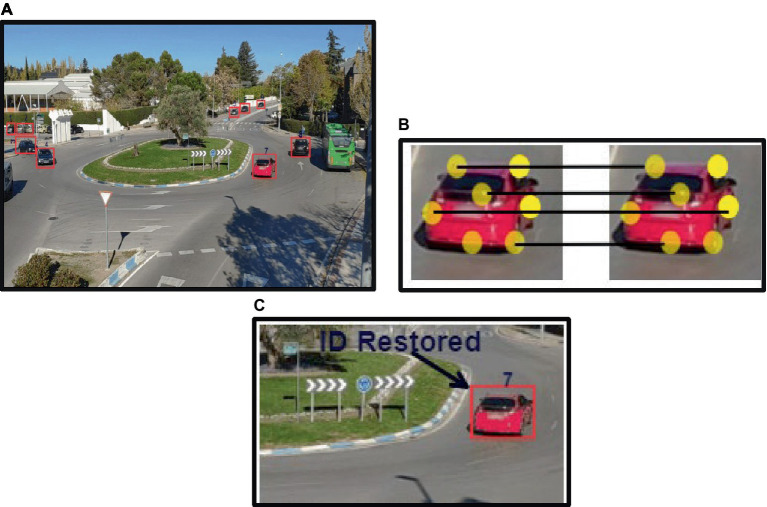
ID assignment and restoration: **(A)** ID assigned to each vehicle based on ORB features; **(B)** features matching across frames; **(C)** ID restored for the same vehicle in succeeding frame.

The identified cars in the subsequent frames were compared using the extracted ORB features, and if a match was discovered, the ID was restored; if not, the vehicle was recorded in the system with a new ID (Cai et al., 2024). ID is restored across frames and ORB feature description is applied to the extracted cars; results are shown in Figure 7.

### 3.6 Vehicle counting

**Figure 8:**
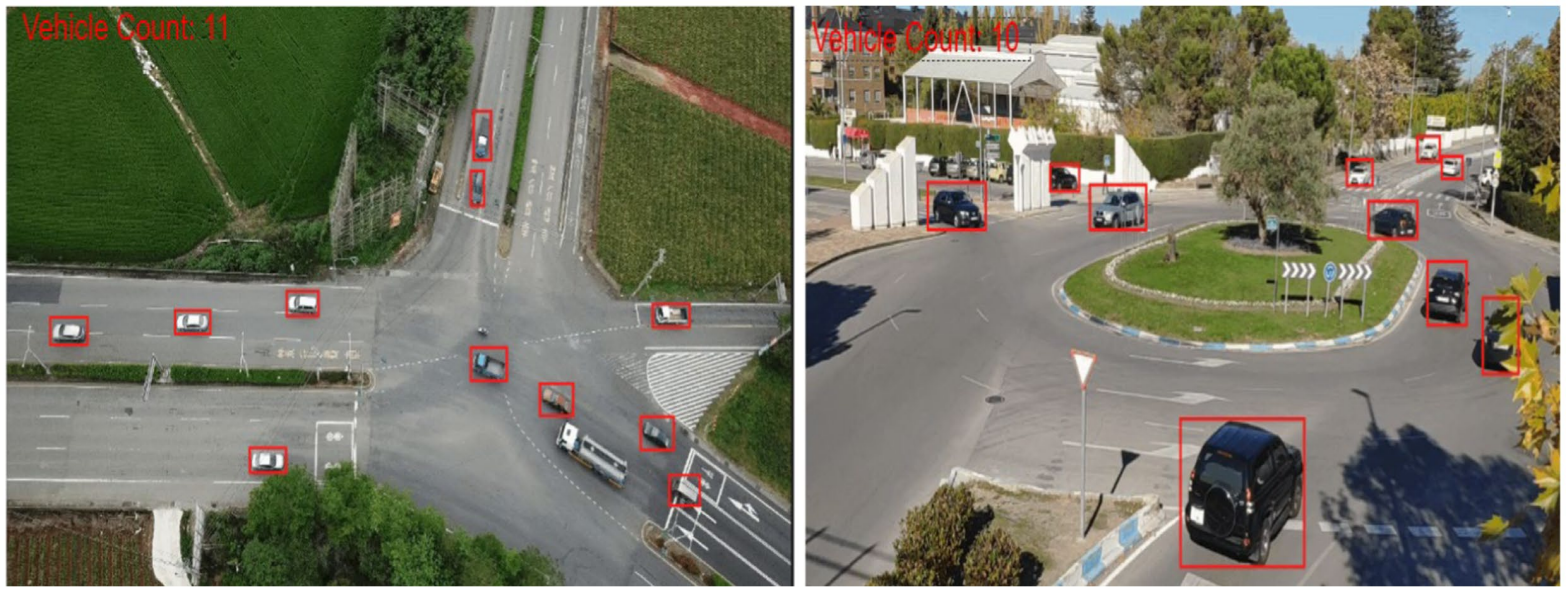
Density estimation by using vehicle count displayed at the left corner of each image.

Using YOLOv8’s vehicle detections, we incorporated vehicle counts in every image frame to conduct a thorough analysis of the traffic situation (Tian et al., 2022; Yang et al., 2023). Using a counter, each seen vehicle was painstakingly recorded under equation. Road traffic density at different times may be measured by counting the number of cars within each frame (Minh et al., 2023). This data is essential for enabling quick responses to unforeseen events like traffic jams or other circumstances that might impair traffic flow (Wu et al., 2019; Peng et al., 2023).


$$\mathrm{Vehicle}\;\mathrm{Count}=\overset{N}{\underset{i=1}{\sum }}T$$

where, *T* denotes the vehicle detections within a single frame, with the corresponding output visualized in Figure 8.

### 3.7 Vehicle tracking

**Figure 9:**
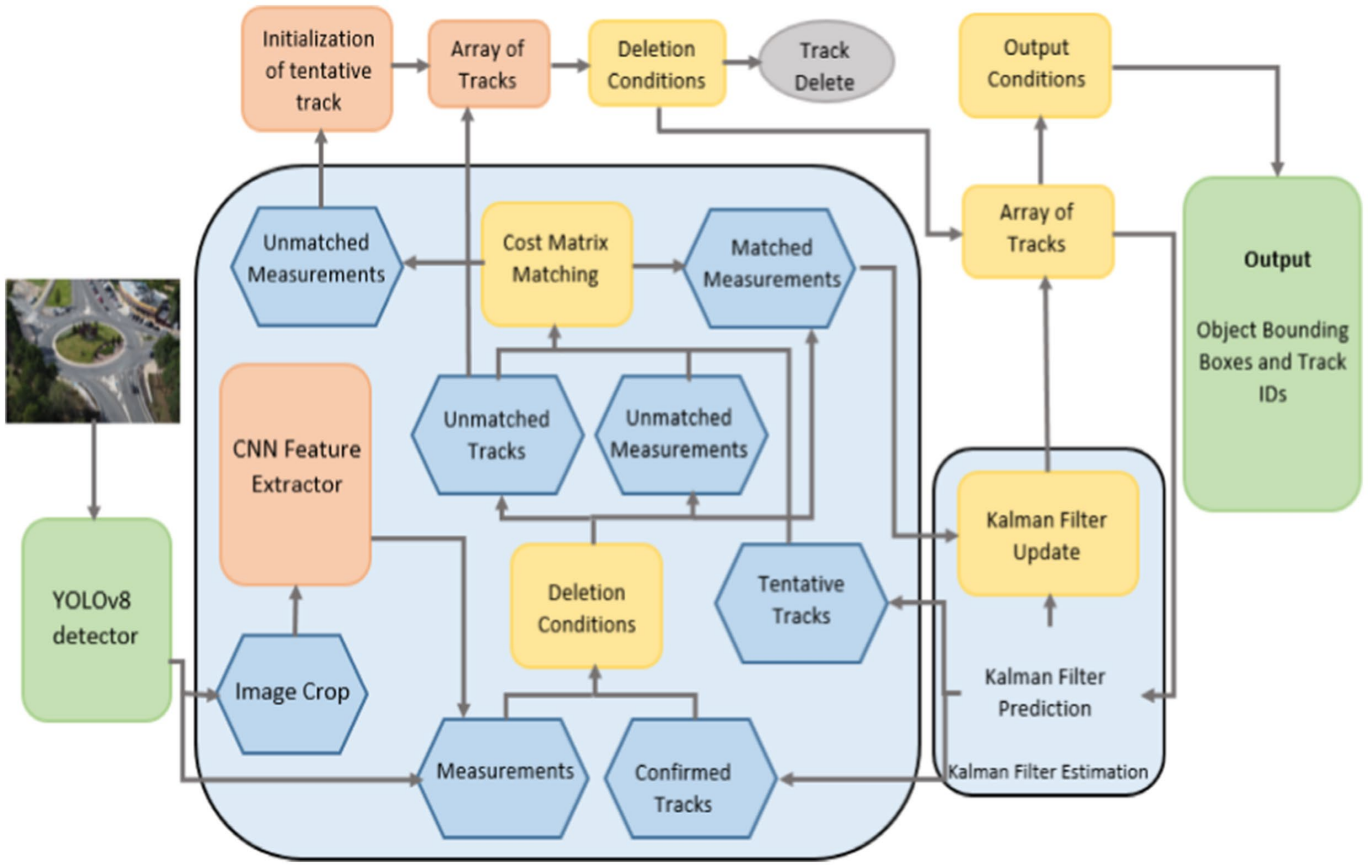
Steps of vehicle tracking using DeepSORT algorithm.

**Figure 10:**
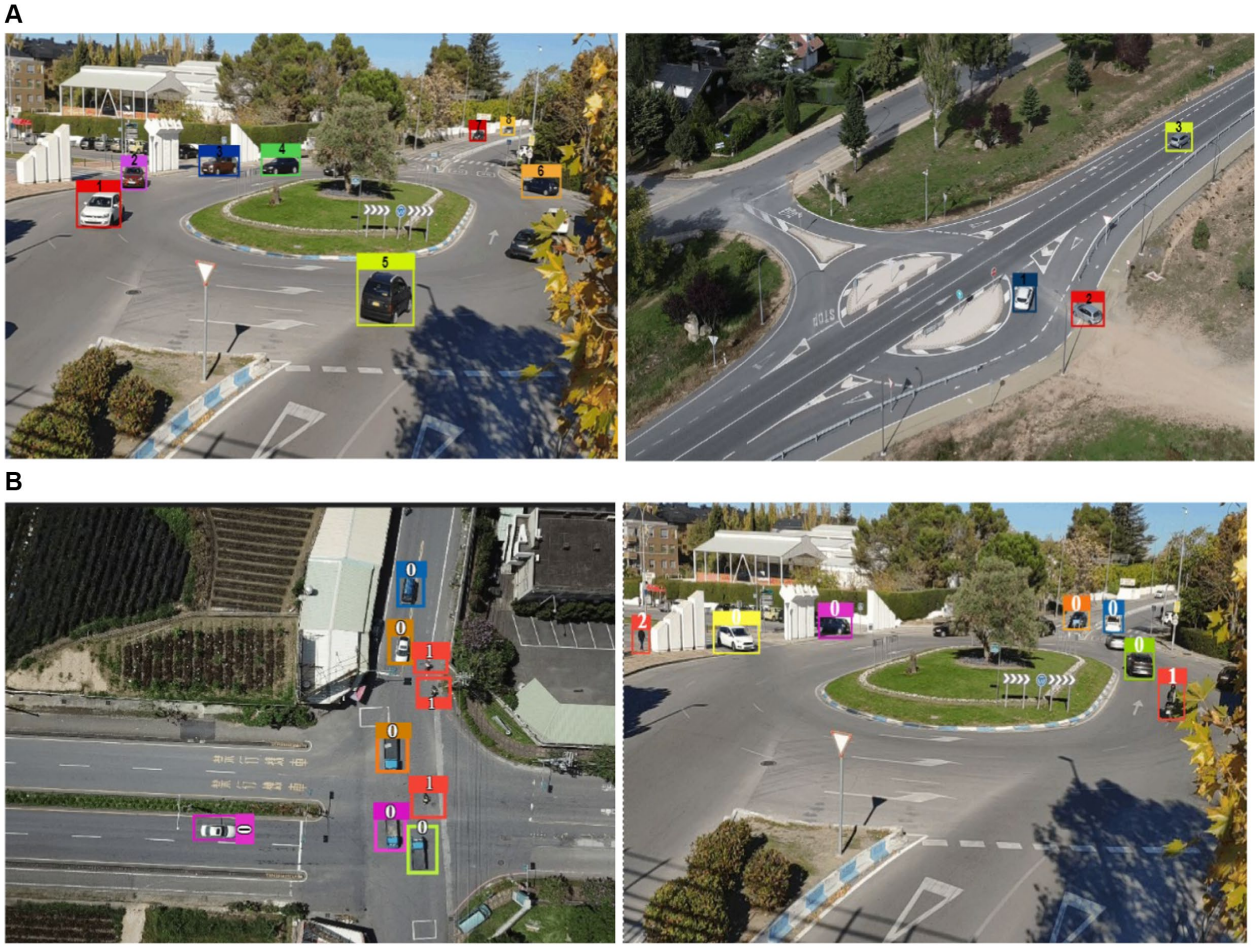
Tracking results using DeepSORT tracker across the image frames **(A)** Vehicle dectection only **(B)**. Multiple-object detection (0 = Vehicles, 1 = Bike, 2 = Pedestrians in frames).

We utilized the DeepSORT tracker to observe the movements of vehicles frame by frame. DeepSORT is a tracking approach that makes use of deep learning characteristics with the Kalman filter to track objects based on their appearance, motion, and velocity (Bin Zuraimi and Kamaru Zaman, 2021; Sun G. et al., 2022). Using the Mahalanobis distance metric between the Kalman state and the freshly obtained measurement, (Li et al., 2018 Sun Y. et al., 2022) the motion information is merged as described in equation:


$$k^{\left(1\right)}\left(i,j\right)={\left(k_{j}-v_{i}\right)}^{T}S_{i}^{-1}\left(k_{j}-v_{i}\right)$$

where k _𝑗_ is the *j*th bounding box detection and (*v_i_, S_i_*) is the *i*th track distribution projection into space measurement. The appearance information has been computed using the smallest cosine distance, as provided by equation, between the *i*th and *j*th detections in appearance space.


$$k^{\left(2\right)}\left(i,j\right)=\min\left\{1-t_{j}^{T}t_{k}^{\left(i\right)}|r_{k}^{\left(i\right)}\in R_{i}\right\}$$

where *t_j_* and $t_{k}^{\left(i\right)}$ represent the appearance and associated appearance descriptor, respectively. The extracted appearance and motion information is combined as given in equation:


$$c_{i,j}=\lambda k^{\left(1\right)}\left(i,j\right)+\left(1-\lambda \right)k^{\left(2\right)}\left(i,j\right)$$

where *c* is the corresponding weight. The appearance features are produced by a pre-trained CNN model that contains two convolution layers, six residual layers linked to a dense layer, one max pooling layer, and l2 normalization (Kumar et al., 2023; Mi et al., 2023). The DeepSORT algorithm’s tracking mechanism is shown in Figure 9 (Singh et al., 2023). In Figure 10, the tracking result is shown.

### 3.8 Vehicle trajectory estimation

In addition to the previously computed density, we approximated the path traveled by each tracked vehicle. The trajectories taken by a vehicle may be utilized to construct vehicle detection (Adi et al., 2018; Bozcan and Kayacan, 2020). It may also be used to identify trajectory conflicts and accidents if it is further developed. The route is plotted if the vehicle is tracked (Chen and Wu, 2016; Wang et al., 2022). To approximate the trajectories, we used geometric coordinates from observed rectangular boxes. DeepSORT was used for location estimation and coordinate retrieval (Leitloff et al., 2014; Sheng et al., 2024). The center points of estimated locations, which represent individual vehicle IDs, were noted on a separate image, and then linked to construct trajectories.

The approach feeds detection coordinates into the DeepSORT tracker, which predicts vehicle placements in the following frame. Vehicle IDs are retrieved using ORB features; if the number of matches exceeds the threshold, relevant IDs are allocated, and new entries are assigned new IDs (Hou et al., 2023b). Rectangular coordinates and midpoints are used to trace vehicle routes. Algorithm 1 provides the exact processes for estimating the trajectory.

#### Trajectory estimation of tracked vehicles
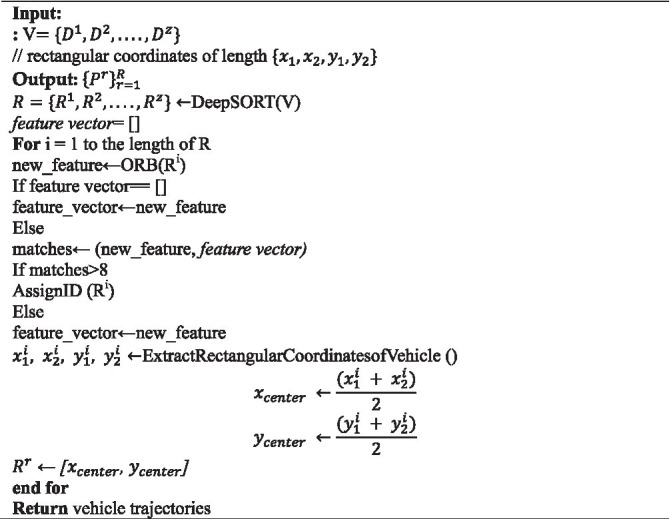


ALGORITHM 1

## 4 Experimental setup and datasets

### 4.1 Experimental setup

PC running x64-based Windows 11, with an Intel Core i5-12500H 2.40GHz CPU, 24GB RAM and other specifications is used to perform all the experiments. Spyder was used to acquire the results. The system employed two benchmark datasets, VEDAI and SRTID, to calculate proposed architecture’s performance. In this section, concise discussion of the dataset used for vehicle identification and tracking system is done, as well as the results of several tests undertaken to examine the proposed system along with its assessment in comparison to numerous existing state-of-the-art traffic monitoring models.

### 4.2 Dataset description

In the subsequent subsection, we provide comprehensive and detailed descriptions of each dataset used in our study. Each dataset is thoroughly introduced, highlighting its unique characteristics, data sources, and collection methods.

#### 4.2.1 VEDAI dataset

The VEDAI dataset (Sakla et al., 2017) is a standard point of reference for tiny target identification, specifically aerial images vehicle detection. This dataset comprises roughly 1,210 images of two distinct dimensions such as 1,024 × 1,024 pixels and 512 × 512 pixels. Both near-infrared and visible light spectra environment photos are acquired in this collection. The cars in acquired aerial shots feature incredibly tiny dimensions, lighting/shadowing shifts, various backdrops, multiple forms, scale variations, and secularities or occlusions. Moreover, it comprises nine separate kinds of automobiles, including aircraft, boats, camping cars, automobiles, pick-ups, tractors, trucks, vans, and other categories.

#### 4.2.2 Spanish road traffic images dataset

The dataset consists of 15,070 images in.png format, followed by an equal number of files with the txt extension containing descriptions of the objects found in each image. There are 30,140 files including images and information. The images were shot at six separate places along urban and interurban highways, with motorways being deleted. The images include 155,328 identified vehicles, including automobiles (137,602) and motorbikes (17,726) (Bemposta Rosende et al., 2022).

#### 4.2.3 VAID dataset

The VAID collection consists of six vehicle image categories: minibus, truck, sedan, bus, van, and automobile. The images were taken at a height of 90–95 meters above the ground by a drone under a variety of lighting circumstances. The photographs, which were captured at a resolution of 2,720 × 1,530 and at a frame rate of 23.98 frames per second, show the state of the roads and traffic at 10 locations in southern Taiwan, encompassing suburban, urban, and educational environments (Lin et al., 2020).

#### 4.2.4 UAVDT dataset

UAVDT dataset: Comprising 80,000 representative frames, the UAVDT dataset (Du et al., 2018) includes UAV imagery of cars chosen from 10-h long recordings. Bounding boxes with up to 14 different attributes (e.g., weather, flying altitude, camera view, vehicle category, occlusion, etc.) completely annotate the photos. Each of the three sets—training, val, and testing consists of 5,000, 1,658, and 3,316 images, all 1,024 × 540 pixels. The photographs from the same video have comparable backdrops, camera viewpoints, and lighting (for those recorded at the same time of day).

### 4.3 Experiment I: semantic segmentation accuracy

The DBSCAN and FCM algorithms were compared and assessed in terms of segmentation accuracy and computational time. DBSCAN requires training on a bespoke dataset, increasing the model’s computing cost as compared to FCM. Furthermore, FCM produced superior segmentation results than DBSCAN, therefore we utilized the FCM findings for future investigation. Table 2 shows the accuracy of both segmentation strategies.

**Table 2:** Accuracies comparison of DBSCAN and FCM segmentation.

Datasets	Segmentations accuracy	
	DBSCAN	FCM
VEDAI	0.65	0.83
SRTID	0.68	0.79
VAID	0.62	0.72
UAVDT	0.65	0.75

### 4.4 Experiment II: precision, recall, and F1 scores

The effectiveness of vehicle detection and tracking has been assessed using these evaluation metrics, namely Precision, Recall, and F1 score as calculated by using equations below:


$$\mathrm{Precision}=\frac{\sum TP\;}{\sum TP+\sum FP}$$

$$\mathrm{Recall}=\frac{TP\;}{TP+FN}$$

$$F1\;\mathrm{Score}=\frac{2\left(\mathrm{Precision}\times \mathrm{Recall}\right)}{\left(\mathrm{Precision}+\mathrm{Recall}\right)}$$

Table 3 shows vehicle detection’s precision, recall, and F1 scores on the segmented images, while Table 4 shows vehicle detection’s precision, recall and F1 scores on the raw images. True Positive indicates how many cars are effectively identified. False Positives signify other detections besides cars, whereas False Negatives shows missing vehicles count. The findings indicate that this suggested system can accurately detect cars of varying sizes.

**Table 3:** Precision, recall, and F1 Score for vehicle detection via YOLOv8 over segmented and raw images.

Datasets	Precision	Recall	F_1_ score
VEDAI (segmented)	0.86	0.84	0.85
SRTID (segmented)	0.84	0.83	0.83
VAID (segmented)	0.85	0.82	0.83
UAVD (segmented)	0.81	0.82	0.81
VEDAI (raw)	0.83	0.80	0.81
SRTID (raw)	0.79	0.81	0.79
VAID (raw)	0.81	0.78	0.79
UAVDT (raw)	0.76	0.77	0.76

**Table 4:** Precision, recall, and F_1_ score for vehicle tracking via DeepSORT.

Datasets	Precision	Recall	F_1_ score
VEDAI	0.87	0.88	0.87
SRTID	0.83	0.82	0.82
VAID	0.88	0.85	0.86
UAVDT	0.84	0.83	0.83

**Figure 11:**
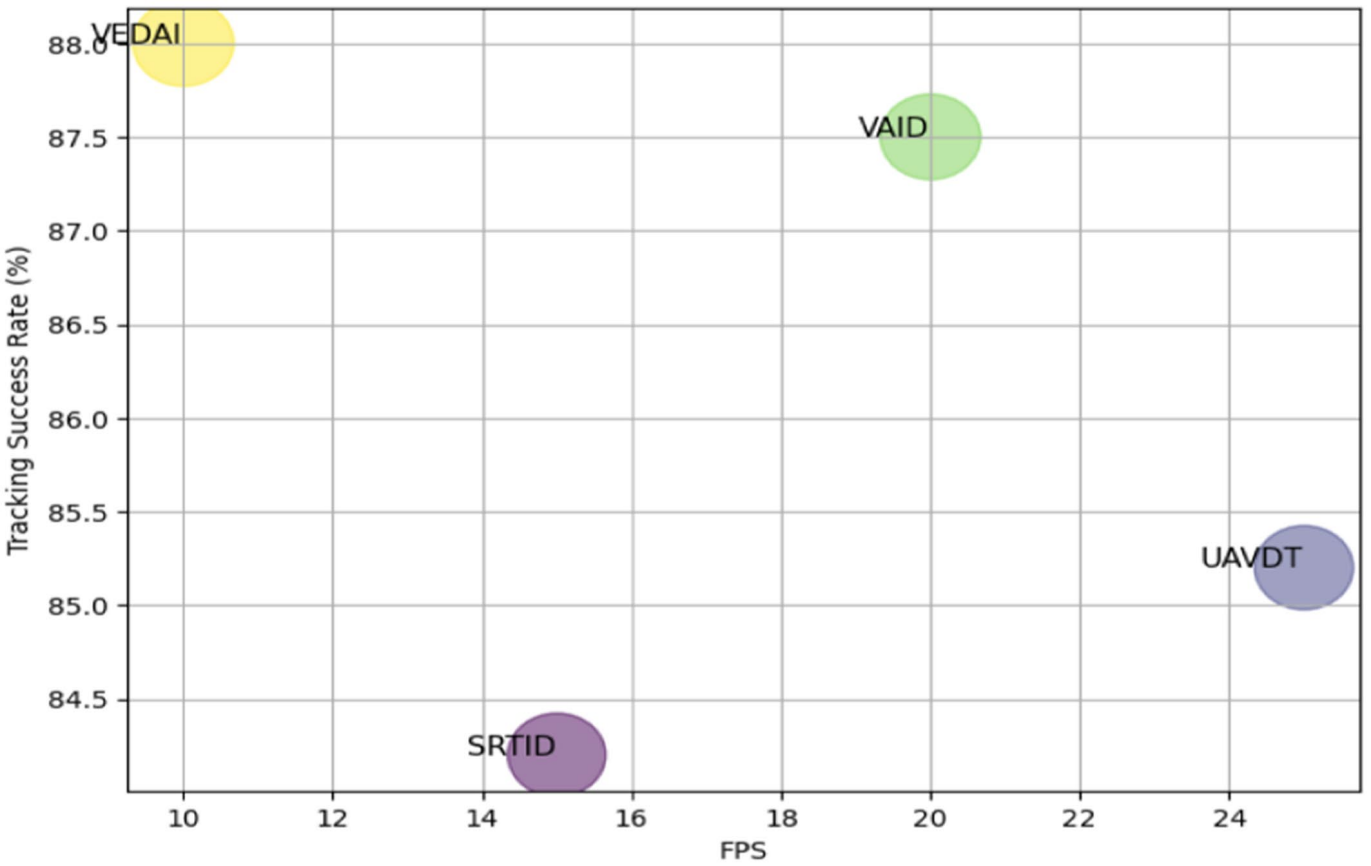
Tracking performance comparison of DeepSORT and ByteTrack across datasets.

In case of tracking, the number of cars successfully tracked is indicated as True Positive, whereas False Positive is the vehicles count falsely recorded, and False Negative represents untracked vehicles count. Table 4 shows the vehicle tracking method’s precision, recall, and F1 scores (Figure 11).

### 4.5 Experiment

#### 4.5.1 ID assignment and ID recovery

We used two new metrics to assess the ID assignment and recovery module, as shown in equations. The AID is the accurate ID rate, which is the proportion of correct ID numbers assigned to automobiles (Table 5).


$$\mathrm{AIDRate}=\frac{\sum_{i=1}^{N}\mathrm{AID}s_{i}}{\sum_{i=1}^{N}ID_{i}}$$

where *N* is the total number of vehicles. $\mathrm{AID}s_{i}$ denotes the overall number of *ID* assignments made to the true vehicles, and $ID_{i}$ denotes all of them. The Recovery Rate represents the percentage of true *IDs* recovered.


$$\mathrm{Recovery}\;\mathrm{Rate}=\frac{\sum_{i=1}^{N}\mathrm{TReCover}s_{i}}{\mathrm{ReCovers}}$$

**Table 5:** Precision, recall, and F_1_ score for vehicle tracking via DeepSORT and ByteTrack.

Datasets	Precision	Recall	F_1_ score	Tracking success rate
VEDAI	0.89	0.90	0.89	88.1%
SRTID	0.85	0.84	0.84	84.2%
VAID	0.90	0.87	0.88	87.5%
UAVDT	0.86	0.85	0.85	85.2%

where the total number of dissimilar vehicles is represented by *N*. $\mathrm{TReCover}s_{i}$ represents the number of true recoveries and ReCovers is the all-existing recoveries (Table 6).

**Table 6:** AIDRate and recovery rate for ID assignment recovery algorithm.

Datasets	AIDRate (%)	Recovery rate (%)
VEDAI	68	65
SRTID	63	59
VAID	59	55
UAVDT	65	60

### 4.6 Experiment IV: vehicle detection and tracking comparison with SOTA models

In this experiment, we have drawn a comparison of proposed model with other popular algorithms. Table 7 represents a comparison between our presented detection algorithm and other methods.

**Table 7:** Accuracy comparison of the proposed approach with SOTA vehicle detection models.

Methods	Accuracy %			
	VEDAI	SRTID	VAID	UAVDT
AVD NET (Mandal et al., 2020)	51.95	62.10	60.75	58.20
YOLOv5 (Hou S. et al., 2023)	75.54	73.20	74.30	72.10
Haar-like features (Nguyen and Tran, 2018)	77.0	65.00	64.50	62.00
D2Det (Cao et al., 2020)	73.40	56.92	68.10	64.30
R-FCN (Zhang, 2020)	68.90	73.0	69.80	70.20
SSD (Smith and Johnson, 2020)	71.00	70.30	81.0	74.50
R-FCN (Kim and Park, 2021)	72.50	70.90	75.0	73.20
NDFT (Cao et al., 2020)	63.50	62.80	64.00	52.03
YOLOv6 (Kumar and Singh, 2023)	74.07	71.20	72.80	74.07
YOLOv7 (Patel and Reddy, 2023)	76.80	74.50	75.60	72.0
Proposed method	79.4	77.7	83.1	77.2

Table 8 depicts the comparison of proposed tracking algorithm. Proposed model model performs better than other state-of-the-art methods.

**Table 8:** Accuracy comparison of the proposed approach with SOTA vehicle tracking models.

Methods	Accuracy %			
	VEDAI	SRTID	VAID	UAVDT
Faster R-CNN (du Terrail and Jurie, 2018)	83.50	78.0	81.0	79.5
Correlation filter tracking (Liu et al., 2019)	76.0	72.5	70.0	74.0
SIFT features (Mu et al., 2016)	72.5	75.10	73.0	71.0
HIOU (Hua and Anastasiu, 2019)	70.0	77.0	69.5	71.5
Kalman filter (Poostchi et al., 2017)	68.0	66.5	65.0	67.5
CNN (Alotaibi et al., 2020)	69.4	71.0	82.0	70.0
Affinity network (Cao et al., 2022)	73.2	74.0	71.5	74.0
MaSiamRPN (Sun et al., 2023)	82.0	79.1	83.0	84.0
Proposed method	88.6	82.2	84.6	86.1

## 5 Discussion/research limitation

For smart traffic monitoring based on aerial images, the suggested model is an efficient solution. While catering to high-definition aerial images, object detection is one of the most difficult problems. To get efficient results, we devised a technique that combines multi-label semantic segmentation with deepsort tracking. However, the suggested technique has significant limitations. First and foremost, the system has only been evaluated with RGB shots acquired during the daytime. Analyzing video or pictorial datasets in low-light conditions or at night can further confirm this proposed technique as a lot of researchers already have succeeded with such datasets. Furthermore, our segmentation and identification system have problems with partial or complete occlusions, tree-covered roadways, and similar items.

## 6 Conclusion

This study presents a novel approach to recognizing and tracking vehicles in aerial image sequences. Before proceeding with the detection phase, the model preprocesses aerial images to remove noise. To decrease complexity, the FCM approach is used for segmentation of all the images. The YOLOv8 algorithm is used for vehicle detection. It identifies vehicles by giving them a unique ID that contains ORB elements to aid recovery. DeepSORT tracks cars across frames and predicts their travel patterns. The suggested approach generated encouraging results across both datasets. The suggested system must be trained with additional vehicle classes. In addition, further elements may be added to increase vehicle recognition and tracking accuracy. In the future, we want to add additional features and dependable algorithms to the proposed model system to boost its efficiency and make it standard for all traffic scenarios.

## Data Availability

Publicly available datasets were analyzed in this study. This data can be found at: https://github.com/mr8bit/vedai https://www.kaggle.com/datasets/javiersanchezsoriano/traffic-images-captured-from-uavs.
